# Integrated framework assessment of water reuse feasibility in mining

**DOI:** 10.1007/s10653-026-03244-2

**Published:** 2026-06-02

**Authors:** João Pedro Machado de Lima, Sonaly Cristina Rezende Borges de Lima, Miriam Cristina Santos Amaral

**Affiliations:** Department of Sanitary and Environmental Engineering, School of Engineering, Federal University of Minas Gerais (UFMG), Av. Antônio Carlos, 6627, Escola de Engenharia, Bloco 1, 4º Andar, Pampulha, Belo Horizonte, Minas Gerais 31270-010 Brazil

**Keywords:** Sustainable mining, Innovation in mining industry, Feasibility of water reuse, Water reuse framework, MCDM in water reuse

## Abstract

Water scarcity, increasing regulatory pressure, and growing societal demands for sustainable resource management have intensified the need for alternative water sources in the mining sector. Treated municipal wastewater represents a potentially viable option; however, its implementation remains constrained by fragmented regulatory frameworks, technical uncertainties, and economic limitations. This study proposes an integrated assessment framework to evaluate the feasibility of treated wastewater reuse in mining operations through a structured approach that operationalizes regulatory alignment, technical compatibility with mineral processing circuits, and economic viability. Unlike conventional binary assessments, this framework introduces a ‘veto logic’ within a tridimensional feasibility space, ensuring that critical technical or legal barriers are not overlooked by simple mathematical averaging. Based on normalized indicators, reuse scenarios are classified into four outcome categories: Fully Feasible, Conditionally Feasible, Economically Constrained, and Structurally Unfeasible. The framework’s utility is demonstrated through a proof-of-concept application using real water quality data, contrasting municipal wastewater reuse against internal acid mine drainage recycling. The results demonstrate that feasibility is highly context-dependent, with external municipal sources often outperforming internal recycling due to lower mineralization and higher regulatory stability. This study contributes a reproducible methodological tool for resilient water management, supporting the reduction of freshwater abstraction and alignment with global sustainability objectives.

**Graphical abstract:**

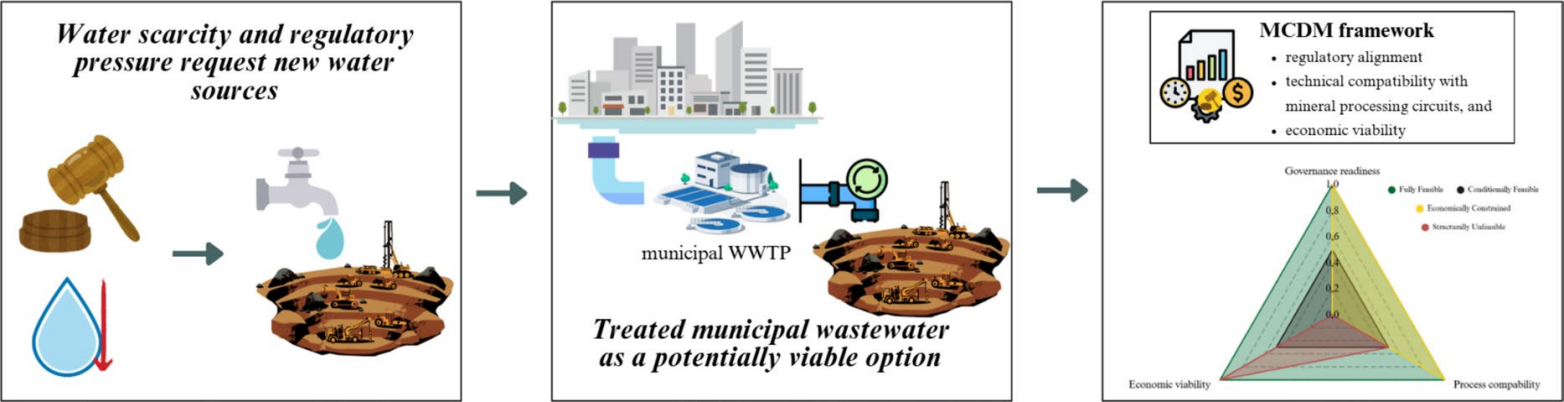

## Introduction

Global population growth and evolving consumption patterns, exacerbated by anthropogenic climate change and erratic precipitation, have intensified freshwater scarcity. This critical scenario necessitates the adoption of alternative water sources and integrated management systems to optimize water-energy-material nexus performance (Procházková et al., 2023). While water reuse (WR) is well-established in agriculture, groundwater recharge, and urban non-potable applications, its integration into heavy industrial processes remains heterogeneous. Reclaiming municipal wastewater offers a strategic pathway for industrial continuity in water-stressed regions. However, despite the global WR market being projected to reach USD 30.5 billion by 2030 (Market Analysis Report, 2025), significant implementation gaps persist.

A fundamental barrier to scaling WR is the spatial mismatch between generating sources—typically centralized Urban Wastewater Treatment Plants (WWTPs)—and industrial end-users. In the mining sector, this challenge is acute, as operations are frequently located in remote or rural areas, far from urban centers (de Lima et al., 2025). Mining is inherently water-intensive; operations such as hydraulic dismantling, dust suppression, and ore transport demand vast volumes. While internal recycling is common, the reliance on external freshwater from rivers and aquifers persists, underscoring the need for a transition toward treated municipal effluents to alleviate pressure on local watersheds (IBRAM & ANA, 2006).

Flotation represents the main water-consuming process in mining (Gunson et al., 2012). Froth flotation is a wet process used to separate valuable minerals from non-valuable ones in the ore, requiring substantial water volumes (Gunson et al., 2012). However, the technical feasibility of using reclaimed water in flotation is governed by complex hydrochemical interactions. Residual organic matter and specific ionic species in treated effluents can non-selectively adsorb onto mineral surfaces, altering hydrophobicity and disrupting reagent-mineral interactions, ultimately compromising recovery efficiency (Jing et al., 2023). While the effects of water quality on flotation are documented (Corin et al., 2024; Levay et al., 2001; Muzinda & Schreithofer, 2018), a systematic method to translate these technical sensitivities into feasibility scores remains absent.

Regulatory landscapes for WR are evolving globally, with robust frameworks established in Europe (EU, 2020/741), North America, and parts of the Middle East (Adamopoulos et al., 2024; de 22 de Octubre, 2024; Decree−Law No.^119^/, 2019, of August 21, 2019; Décret No, 2023–835, 2023; Real Decreto, 1085/2024; Water Substitution & Reuse Policy, 2016), including options beyond industrial applications. Additionally, other countries also have guidelines for water reuse, such as the United States and Canada (Canadian Guidelines for Domestic Reclaimed Water for Use in Toilet & Urinal Flushing, 2010; U.S. Environmental Protection Agency, 2012). Nevertheless, these regulations predominantly target agricultural irrigation and urban utilities, leaving a normative vacuum for mining-specific applications. This absence of targeted standards, coupled with the spatial and technical constraints previously discussed, creates a fragmented decision-making environment where regulatory compliance, technical viability, and economic cost are evaluated in isolation. Furthermore, the transition toward sustainable water management in mining cannot be decoupled from the broader Environmental, Social, and Governance (ESG) risk landscape (Maybee et al., 2023). As mining operations are long-life projects, non-technical risks and ESG issues become increasingly critical over the asset’s life cycle (Maybee et al., 2023). Integrating reclaimed water involves navigating complex socio-political dynamics to maintain a Social License to Operate (SLO), which is not a static agreement but an evolving social contract shaped by diverse stakeholder narratives and power dynamics (Rodolaki et al., 2025).

Despite the growing body of literature addressing water reuse (Campo et al., 2023; Chhipi-Shrestha et al., 2017; Lebron et al., 2023; Rubio et al., 2007; Wade Miller, 2006) and the existence of regulatory instruments in multiple jurisdictions (Adamopoulos et al., 2024; Decree−Law No.^119^/, 2019, of August 21, 2019; Décret No, 2023–835, 2023; Joint Ministerial Decree (JMD) 145116/11, 2011; Real Decreto, 1085/2024, de 22 de Octubre, 2024; The Environmental Law, 1994; Oron et al., 2014; United Arab Emirates, 2021), decision-making regarding reclaimed water integration into mining operations remains fragmented. Existing assessments typically evaluate regulatory compliance, technical compatibility, or economic feasibility in isolation. However, WR in mineral processing systems is intrinsically multidimensional, requiring simultaneous alignment among governance structures, process sensitivity, spatial configuration, and lifecycle costs. The absence of an integrated analytical framework capable of systematically articulating these dimensions constitutes a critical gap in the current literature.

Several analytical frameworks have been proposed to evaluate water reuse, industrial symbiosis, circular water management, and integrated resource systems (Asano & Mills, 1990; Campo et al., 2023; Chhipi-Shrestha et al., 2017; Hejazi et al., 2014; Neumann et al., 2024; Vidotti et al., 2024; Wade Miller, 2006; Wilcox et al., 2016). These models commonly emphasize environmental performance indicators, lifecycle assessment (LCA), techno-economic analysis (TEA), or integrated water resources management (IWRM) principles. While these approaches provide valuable insights into sustainability metrics and resource efficiency, there is no integrated framework capable of articulating the multidimensional trade-offs between governance, process chemistry, and lifecycle costs.

To bridge this gap, this study develops a structured Multi-Criteria Decision-Making (MCDM) framework specifically designed to assess the feasibility of treated municipal wastewater reuse in mining. The proposed model is operationalized through a scoring matrix and validated via a worked example. Specifically, this research addresses: (i) the identification of key determinants for reuse feasibility; (ii) the structural integration of these determinants into a decision-support tool; and (iii) the framework’s capacity to consistently classify scenarios under diverse technical and spatial conditions.

## Methodology

The identification of regulatory frameworks was conducted through a semi-systematic scoping approach, focused on capturing the diversity of global water reuse policies applicable to industrial and mining contexts. The search was performed between August/2025 and December/2025 without temporal restrictions to ensure a comprehensive historical and contemporary legal mapping. The primary data source was the FAOLEX Database (Food and Agriculture Organization of the United Nations), complemented by the U.S. EPA REUSExplorer Tool (USEPA, 2024) for technical and economic standards. The search strategy utilized combinations of Boolean operators and keywords, including: ‘water reuse’, ‘water reuse in industry’, ‘water reuse in mining’, and ‘wastewater treated reuse’. The initial search yielded 24 distinct regulatory instruments from the Aqualex/FAOLEX platform (FAOLEX, 2026). Each document underwent a qualitative content analysis to extract information for the ‘Governance’ and ‘Regulatory’ dimensions of the proposed framework. The regulatory analysis covered 21 countries and one supranational framework (European Union), representing a diverse range of hydrological, institutional, and economic contexts.

Following document retrieval, legal instruments were screened to identify provisions explicitly addressing reclaimed water quality criteria, permitted end uses, monitoring requirements, industrial reuse authorization, and governance structure. Only legally binding instruments (laws, decrees, binding resolutions, or enforceable standards) were included in the comparative analysis. Particular attention was given to identifying whether regulatory frameworks explicitly address industrial reuse in mineral processing or if standards are primarily designed for agricultural and environmental applications. Retrieved regulations were categorized according to (i) scope of application (agricultural, urban, environmental, industrial reuse); (ii) type of regulatory instrument; (iii) water quality parameters regulated; (iv) presence of industrial-specific reuse criteria; and (v) institutional responsibility and enforcement structure. This stage enabled the identification of governance gaps, fragmentation patterns, and the absence or presence of harmonized industrial reuse standards.

To complement the regulatory analysis, a technical evaluation was conducted focusing on the compatibility between wastewater characteristics and mineral processing requirements. The main observed points were: (i) review of physicochemical parameters typically regulated in reuse legislation (e.g., BOD, TSS, turbidity, microbiological indicators); (ii) identification of parameters critical to mineral processing performance (e.g., ionic strength, sulfate concentration, hardness, dissolved metals, residual organics); (iii) analysis of sensitivity of major unit operations, particularly flotation circuits, to variations in water chemistry; and (iv) consideration of accumulation effects in closed-circuit recirculation systems. The objective was to determine whether regulatory compliance ensures operational compatibility or whether a regulatory–technical gap persists.

An economic dimension was incorporated to evaluate the practical viability of reclaimed water integration into mining operations. This included (i) identification of capital expenditures (CAPEX), such as pipeline infrastructure, pumping stations, equalization reservoirs, polishing systems, and circuit adaptations; (ii) identification of operational expenditures (OPEX), including pumping energy (distance and elevation dependent), chemical conditioning, monitoring, maintenance, and risk management; (iii) assessment of spatial mismatch between centralized municipal wastewater treatment plants and remote mining sites; and (iv) spatial configuration (distance and elevation gradient) was treated as a critical variable influencing lifecycle costs and implementation feasibility.

Based on the integrated findings from the regulatory analysis, technical compatibility assessment, and economic evaluation, a conceptual framework was developed. The framework is a Multi-Criteria Decision-Making method, commonly applied in environmental management, due to its characteristics of permitting to deal with multifaceted and uncertain decision (Najafi et al., 2025). The framework suggested was constructed through an inductive synthesis process, whereby recurring constraints and enabling conditions identified across jurisdictions and technical contexts were organized into three analytical dimensions, components of a Multi-Attribute Decision-Making (MADM), one of the two MCDM alternatives. The structural development of this MCDM framework draws inspiration from recent advancements in transitional and post-mining land-use evaluations, which emphasize the need to move beyond rigid, pre-structured problems toward adaptable, scenario-based classifications (Amaro et al., 2022). By organizing the analytical dimensions into explicit criteria and subcriteria, this approach allows for the transparent incorporation of both technical complexities and stakeholder preferences, ensuring that the ordinal scoring logic is empirically grounded and methodologically robust (Amaro et al., 2021).

To operationalize the conceptual framework into a practical MCDM tool, specific evaluation criteria and scoring thresholds were established for each analytical dimension. Each sub-criterion is evaluated on a normalized ordinal scale of 0.0 (low feasibility or high barrier), 0.5 (moderate feasibility or barrier that can be overcome), and 1.0 (high feasibility or ideal condition). This discretized scaling approach allows transparent positioning of scenarios within the tridimensional feasibility space while preserving interpretative clarity and minimizing artificial precision. The categorical thresholds were defined based on qualitative and quantitative criteria specific to each dimension, ensuring internal consistency in the MCDM structure. The integrated framework is shown in Table 1.

**Table 1:** Matrix of criteria and scoring for assessing the feasibility of reuse

Dimension	Subcriteria	Definition	Low (0.0)	Medium (0.5)	High (1.0)
Governance and institutional readiness	Existing standards	Evaluates the presence and clarity of legal frameworks governing treated wastewater reuse in industrial or mining settings	No regulation exists, or the norm explicitly prohibits the use of treated effluent in industrial environments	General norms exist, but quality standards for industrial use are ambiguous or excessively restrictive	Specific and clear legislation for industrial reuse exists, with well-defined and achievable technical parameters
	Licensing complexity	Assesses the administrative burden, time, and institutional coordination required to obtain reuse permits and SLO	Uncertain licensing process involving multiple uncoordinated agencies; low acceptance by the local community; estimated time > 2 years	Licensing is possible but requires complex environmental impact studies, medium acceptance by the local community and extensive long-term monitoring	Simplified or well-established licensing process, with clear deadlines and precedents in the jurisdiction, and easy acceptance by the local community
Process compatibility and technical feasibility	Water availability	Assesses the capability of the WWTP to meet the volume required by the mining operations continuously	Intermittent supply or covers < 30% of the mine’s demand	Stable supply, covering 30–70% of the demand	Continuous supply, covering > 70% of the demand
	Water quality compatibility	Evaluates the degree of compatibility between the STS effluent and the specific requirements of the mineral processing	Contains contaminants (e.g., specific ions, organics) that degrade the process	Requires polishing treatment (e.g., advanced oxidation, membranes) to remove specific interferents	Meets technical requirements without post-treatment
Economic viability	Location (Distance)	Evaluates the geographical distance between the WWTP and the mine, which directly impacts pumping and infrastructure costs (CAPEX/OPEX)	Distance > 50 km, requiring prohibitive water adduction CAPEX	Distance between 10 and 50 km, requiring public–private partnerships or shared infrastructure to be viable	Distance < 10 km, minimizing infrastructure and pumping costs
	Opportunity cost	Compares the total cost of using reclaimed water (tariff+OPEX) against the current cost of freshwater abstraction or scarcity penalties	Reclaimed water cost is significantly higher than the current freshwater cost or scarcity fines	Cost is equivalent to conventional water, but offers greater water security during drought periods	Reclaimed water cost is lower than the total cost of freshwater abstraction (including abstraction fees and raw water treatment)

The aggregate score for each core dimension is calculated as the arithmetic mean of its respective sub-criteria (Governance—Dg, Technical—Dt, and Economic—De). All three dimensions were considered equally weighted (w = 1/3) due to the absence of stakeholder-specific preference elicitation. While a global Feasibility Index (Fi) can be estimated as the unweighted average of the three dimensions (Eq. 1), water reuse projects are typically constrained by specific operational or legal bottlenecks rather than poor average performance.

1 $$ Fi = {{\left( {Dg + Dt + De} \right)} \mathord{\left/ {\vphantom {{\left( {Dg + Dt + De} \right)} 3}} \right. \kern-0pt} 3} $$

The resulting model provides a structured decision-support tool capable of classifying reclaimed water reuse scenarios into feasibility categories based on institutional alignment, operational stability, and financial competitiveness, as presented in Table 2.

**Table 2:** Classification of the feasibility index (Fi) and project implementation outlook

Category	Project outlook and decision support	Fi range
Structurally unfeasible	Implementation is currently blocked by fundamental barriers. High risk of regulatory non-compliance or technical failure. Significant structural changes in the legal framework or massive technological breakthroughs are required	0.0 ≤ Fi ≤ 0.3
Economically constrained	The project presents high implementation risks. While some dimensions may be viable, the lack of economic competitiveness or infrastructure gaps makes it unattractive without substantial subsidies or external partnerships	0.3 < Fi ≤ 0.6
Conditionally feasible	The project is viable but requires targeted optimization. Success depends on specific technical adjustments (e.g., polishing treatments), site-specific licensing negotiations, or optimization of logistics to improve the cost–benefit ratio	0.6 < Fi ≤ 0.9
Fully feasible	Ideal conditions for immediate implementation. High alignment between technical requirements, legal security, and economic returns. Minimal adaptation of existing systems is required	0.9 < Fi ≤ 1.0

A critical constraint is applied to this numerical synthesis: if any single dimension (Di) receives a score of 0.0, the project is automatically downgraded to the ‘Unfeasible’ category, regardless of the aggregate Fi. This ‘veto’ logic ensures that fundamental barriers, such as absolute regulatory prohibition or technical incompatibility, are not masked by high performance in other dimensions.

## Mining wastewater characteristics

Mining wastewater is inherently complex and highly variable. Unlike municipal effluents, which present relatively predictable compositions, mining effluents are strongly influenced by mineralogy, oxidation reactions, reagent usage, solid–liquid separation processes and the operational water management strategy adopted at each site. As a result, their physicochemical profile may fluctuate significantly both spatially and temporally within the same operation. The combined presence of acidity, dissolved metals, high salinity, suspended solids, and residual reagents makes mining wastewater qualitatively different from most conventional industrial and municipal effluents. Consequently, any assessment of water reuse feasibility must begin with a clear understanding of these intrinsic characteristics, as they directly determine treatment requirements, operational compatibility, and economic implications.

To illustrate the variability and magnitude of these parameters across different mining contexts, Table 3 compiles reported physicochemical characteristics of wastewater from copper, coal, pyrite, and gold mining operations, providing the basis for the subsequent integrated feasibility assessment.

**Table 3:** Reported water quality characteristics of mining effluents and process waters categorized by mineral type. *Source*: Compiled by the authors based on literature review (2026)

	pH	EC (µS/cm)	TDS (mg/L)	TSS (mg/L)	SO_4_^2−^ (mg/L)	Key metallic species (mg/L)	Ref
Process water and waste							
Circuit water	6.7	NR	9.90	NR	14.42	Ca (120); Fe (6.16); K (145); Na (4.35); Ni (4.15)	(Levay et al., 2001)
Process wastewater-Gold	9.80	4560	NR	NR	NR	Turbidity (16,400 NTU)	(Falconi et al., 2023)
Tailings dam-Iron	8.80	840	NR	NR	NR	NR	(Falconi et al., 2023)
Tailings dam-Copper	7.57	NR	NR	28	NR	Turbidity (11 NTU)	(Falconi et al., 2023)
Flotation tailings-Iron	9.12	NR	NR	178	NR	NR	(Falconi et al., 2023)
Flotation wastewater (bottom)-Copper	7.95	NR	NR	825	NR	Turbidity (961 NTU)	(Falconi et al., 2023)
Decant pond return	6	NR	11	NR	17.08	Ca (135); Cl⁻ (2.21); K (150); Mg (2.25); Na (4.54)	(Levay et al., 2001)
Settling tanks-Gold mining	NR	NR	NR	NR	NR	Cu (202.9); Fe (916.5); Mg (102.8); Ni (239.8)	(Moreira et al., 2024)
Settling tanks-Gold mining	2.13	NR	NR	NR	NR	Cu (211.1); Fe (1335.2); Mg (443.94); Mn (149.4); Ni (227.83)	(Moreira et al., 2024)
**AMD-Copper mining**							
AMD 1st stage-Copper	2.98	16,800	NR	NR	NR	Al (627.6); Cu (398.2); Fe (2230.6); Zn (2074.5)	(Macías et al., 2017)
AMD 2nd stage-Copper	3.64	5700	NR	NR	NR	Al (15.3); Fe (13.7); Mg (617); Mn (112.9)	(Macías et al., 2017)
**AMD-Coal mining**							
AMD-Brown coal	3.5	NR	NR	NR	1375	Al (19.8); Ca (296.6); Fe (53.5); Mn (6.2)	(Heviánková et al., 2014)
AMD-Coal (General)	5.79	NR	NR	NR	2870	Al (6.0); Ca (470); Fe (9.0); Na (3061); Zn (2.8)	(Galloux et al., 2015)
AMD-Coal (High metal)	2.33	7.79	NR	NR	7410.4	Al (269.3); Fe (611.3); Mn (37.9); Zn (62.6)	(Carneiro Brandão Pereira et al., 2020)
**AMD-Pyrite mining**							
AMD 1st stage-Pyrite	2.70	NR	NR	NR	6883	Al (117); Fe (3150); Mn (27.7); Zn (4.9)	(Chen et al., 2015)
AMD 2nd stage-Pyrite	2.50	NR	NR	NR	7931	Al (1878); Fe (3580); Mn (145.7); Zn (80.7)	(Chen et al., 2015)
AMD-Pyrite (general)	2.70	NR	NR	NR	11,700	Al (251); Fe (744); Mn (467); Zn (976)	(Ayora et al., 2016)
AMD-Pyrite (general)	2.10	NR	NR	NR	NR	Al (251); Fe (1141); Mn (115); Zn (78)	(López et al., 2021)
**AMD-Gold mining**							
Pressure Oxidation (POX)	1.37	16,800	NR	NR	NR	Al (484.6); Cu (171.5); Fe (829.2); Mg (2662.1)	(Lebron et al., 2023)
AMD 1st stage-Gold	3.76	2573	3025	NR	1959.5	Ca (284); Cl⁻ (151.5); Mg (226); Zn (3.0)	(A. Aguiar et al., 2018)
AMD 1st stage-Gold	2.74	2744	2839	NR	1406.1	Ca (323); Cl⁻ (54.5); Mg (97)	(A. O. Aguiar et al., 2016)
AMD 2nd stage-Gold	3.35	2965	3398	NR	2767.7	Ca (367); Cl⁻ (14.1); Mg (299); Zn (0.36)	(A. Aguiar et al., 2018)
AMD 2nd stage-Gold	3.76	2573	30,925	NR	1959.5	Ca (284); Cl⁻ (151.5); Mg (226)	(A. O. Aguiar et al., 2016)
AMD-Gold (general)	4.15	NR	NR	NR	1123	Al (3); Cu (0.1); Fe (12); Mg (170); Mn (1331); Zn (1.4)	(Shabalala et al., 2017)

EC–Electric conductivity; TDS–Total Dissolved Solids; TSS–Total Suspended Solids; Ref–Reference; NR–Not reported

While the central focus of this research is the feasibility of integrating treated municipal wastewater as an external supply source, Table 3 provides an essential baseline of internal water quality for comparison. It offers an overview of water quality parameters associated with various internal mining operations, including copper, iron, gold, coal, and pyrite mining. Considering reuse application, the main parameters are metals, conductivity, TDS/TSS, and pH, due to the influence they have in salinity, hardness and behavior of flotation reagents.

Contrasting the relatively stable profile of municipal effluents with internal mining streams is crucial. Parameters such as electrical conductivity (EC), total dissolved solids (TDS), sulfate (SO_4_^2−^), chloride (Cl^−^), and major cations (Na^+^, Ca^2+^, K^+^) are essential to determine the ionic load of effluents and their compatibility with reuse systems. High Electric conductivity values (e.g., 16,800 µS/cm in gold mine wastewater under POX) suggest elevated salinity, which could impair equipment through scaling or corrosion and limit reuse in processes sensitive to ionic strength (Guo et al., 2023). The pH values of mining wastewater range widely, varying from acidic conditions (pH 3.6 in drainage water from copper flotation pits) to alkaline levels (up to pH 9.8). Such variability demands pH correction prior to reuse, particularly when water is intended for equipment cooling, reagent preparation, or dust suppression. High values of TSS and turbidity were observed in flotation-related effluents, such as 825 mg/L TSS and 961 NTU in copper wastewater. These values are far above acceptable thresholds for most reuse applications, implying that sedimentation, filtration, or advanced clarification would be required before reuse. Such particulate-rich effluents may also carry adsorbed heavy metals or residual reagents, which further complicate treatment.

Although sparsely reported, key trace metals such as Fe, Ni, Cu, and Zn are crucial for assessing reuse safety and regulatory compliance. For instance, Fe concentrations of 6.16 mg/L and Ni at 4.15 mg/L (in circuit water) are considerably elevated compared to typical reuse standards (e.g., < 1 mg/L for Fe in industrial cooling) (EMSD, 2016). These metals are known to be toxic, can accumulate in systems, and interfere with treatment and industrial processes (Buecker & Kalakodimi, 2021). The absence of consistent data on Zn and Cu suggests the need for more comprehensive monitoring.

Copper mining effluents exhibit consistently poor quality, characterized by low concentration on pH (ranging from 2.0 to 3.6, except one single sample of 6.0), and significant metal loads—copper (up to 398.2 mg/L), zinc (up to 2074.5 mg/L), and iron (up to 2230.6 mg/L). As brought before, the iron limit concentration for fresh water-cooling tower is 1.0 m/L (EMSD, 2016), while the recommended maximum concentrations of trace elements in irrigation water are 1.0 mg Cu/L in Italy, 2.0 mg Zn/L, and 5.0 mg Fe/L in USA (Jeong et al., 2016). Moreover, these waters display elevated conductivity (up to 16,800 µS/cm) and substantial concentrations of dissolved ions such as sodium, magnesium, and sulfates, suggesting a strong requirement for desalination and metal removal. Given these conditions, reuse is not feasible without extensive treatment involving neutralization, precipitation, adsorption, and filtration processes, as widely explored by Y. Liu et al. (2023).

In contrast, coal mining waters present a more heterogeneous profile, with pH ranging from moderately acidic to near-neutral (2.3–7.34). Conductivity values vary significantly, reaching up to 7410.4 µS/cm in some samples. Some samples still exhibit concerning levels of iron (up to 611.4 mg/L) and sodium (over 3000 mg/L). Despite these issues, coal mine waters require typically pH correction and iron removal, which could be achieve by passive treatment methods (Hedin, 2008; Watzlaf et al., 2004), making them more suitable for reuse in industrial or operational contexts where water quality standards are less stringent.

Pyrite mining effluents show some of the most extreme water quality conditions. These samples are highly acidic (pH 2.1–2.7) and heavily contaminated with metals such as iron (up to 1141 mg/L), manganese (up to 467 mg/L), and zinc (up to 976 mg/L). Salinity is also excessive, with conductivity reaching 11,700 µS/cm and sulfate concentrations often surpassing 7000 mg/L. These results reflect the intense oxidation of sulfide minerals and the mobilization of secondary pollutants. Due to the combined impact of high acidity, salinity, and metal content, reuse of pyrite effluent is extremely limited without robust, multi-stage treatment technologies (Ding et al., 2026).

Water from gold mining operations presents the most variable quality among the mining types analyzed. The pH of these samples ranges from highly acidic (1.37) to moderately acidic (4.15), with corresponding increases in conductivity (up to 29,065 µS/cm). Concentrations of aluminum, copper, and zinc vary significantly, with some samples reporting up to 484.65 mg/L of Al, 171.57 mg/L of Cu, and 199 mg/L of Zn. The heterogeneity of gold mining waters suggests that while some samples may be amenable to reuse following targeted treatment (e.g., neutralization, TOC removal), others will require intensive and costly processes before being deemed suitable (Gaviria et al., 2024; Lebron et al., 2023; Moreira et al., 2024; Ricci et al., 2017).

The chemical profile of mining wastewater above presented does not only dictate technical feasibility but serves as the primary driver for the Governance dimension (Dg) of the proposed framework. Regulatory complexity is directly proportional to the hazard level of the effluent; for example, water containing heavy metals or toxic reagents triggers more stringent licensing requirements and higher monitoring frequencies than saline-only water. Thus, the characterization phase informs the governance analysis by identifying which legal thresholds (discharge vs. reuse standards) apply, ultimately determining the ‘Regulatory Compatibility’ score within the framework. In summary, the wastewater characteristics described in this section provide the input data for the proposed framework: they define the technical effort required (Dt), dictate the legal pathway and permitting rigor (Dg), and establish the cost baseline for the project’s financial appraisal (De). Any proposal for reclaimed water integration into mining systems must consider not only water quality parameters but also governance frameworks, technical feasibility, and economic viability, as examined in the following sections.

## Governance challenges

Environmental and public health risks are the primary concerns associated with wastewater treatment. The most effective way to regulate minimum water quality requirements for reuse is through legislation. Plans, programs, guidelines, and frameworks are usually developed to help those involved in upgrading technologies to achieve the required quality for the intended use. The Governance dimension (Dg) evaluates the alignment between the water reuse project and the existing legal and institutional frameworks. Unlike technical or economic variables, governance factors often act as structural constraints; a project may be technically feasible and economically attractive but remain unfeasible due to regulatory prohibitions or the absence of specific standards for the mining sector.

While regulatory compliance provides a legal baseline, it does not guarantee public acceptance (Saenz, 2022). A comprehensive assessment must embed ESG risk evaluation to map the specific fears and expectations of diverse stakeholder groups (such as local communities, businesses, policymakers, and environmental activists) prior to project implementation (Paat et al., 2021). Empirical evidence shows that while developers or business sectors may focus on governance or economic risks, local communities predominantly prioritize environmental and direct social impacts (Paat et al., 2021; Saenz, 2018). Therefore, the Governance dimension must also reflect the capacity of the regulatory framework to facilitate procedural fairness and trust, which are foundational for securing and maintaining the SLO.

To ensure a precise assessment, the Governance dimension distinguishes between three regulatory pathways within the mining context:

- Treated municipal wastewater as an external supply source: The primary focus of this framework, governed by cross-sectoral standards and municipal-industrial agreements.
- Internal recycling of process water: Regulated primarily by operational safety and site-specific environmental management.
- Reuse of treated AMD: Subject to stringent environmental liability and discharge laws, where the regulatory burden is significantly higher due to the hazardous nature of the effluent.

Even though water reuse is not a new strategy around the world, the first legislation to bring attention to this topic was launched in 1918 in California, USA (U.S. Environmental Protection Agency, 2012). Years later (1966), Iowa regulated 567 IAC Chapter 62: Effluent and Pretreatment Standards: Other Effluent Limits or Prohibitions. Some years later, on a global level, the World Health Organization (WHO) elaborated the first guideline focused on water reuse in agriculture (Shoushtarian & Negahban-Azar, 2020). This has helped other countries to develop regulations and regulatory guides on the subject (Vidotti et al., 2024).

Since then, a multitude of decrees and guidelines have been established worldwide in the last few years aiming to standardize water or wastewater reuse, including management strategies to account and mitigate risks. The International Organization of Standardization (ISO) has a technical committee, ISO/TC 282, which standardizes water reuse of any type and for any purpose. It covers both centralized and decentralized water reclamation or on-site water reclamation, and direct and indirect reuse applications, considering the potential for unintentional exposure or ingestion (ISO Technical Committees, 2025).

The European Parliament and of The Councils established a framework for Community action in the field of water policy, on October 23rd, 2000, through the Directive 2000/60/EC. This Directive seeks to preserve and enhance the aquatic environment within the Community, with a primary focus on water quality. While quantity control is a secondary consideration, it plays a crucial role in maintaining water quality. Therefore, measures addressing water quantity should also be implemented to support this objective (Regulation (EU) 2020/741 of the European Parliament and of The Council of 25 May 2020, 2020).

Regulation (EU) 2020/741 of the European Parliament and of the Council of 25 May 2020 standardizes minimum requirements for water reuse. The main category dedicated to applying water reuse is agricultural irrigation. The legislation allows other uses, such as industrial water reuse, amenity-related and environmental purposes (European Commission, 2020). However, the Commission Notice, 2022/C 298/01, which provides guidelines to support the application of Regulation 2020/741, specifies water quality requirements exclusively for agricultural irrigation. It does not offer technical guidance or quality standards for reclaimed water for urban or industrial reuse (Commission Notice, 2022/C 298/01, 2022).

In Portugal, Decree-Law 226-A/2007 establishes the regulatory framework for the use of water resources, defining that treated wastewater must be reused whenever possible or appropriate, particularly for irrigating gardens, public spaces and golf courses, in line with the guidance provided for in Directive 91/271/EC (Decree-Law No. 226-A/2007, of 31 May, 2007). Decree-Law 119/2019 in Portugal defines the legal regime for producing water for reuse (WFR) from treated wastewater. It allows WFR from on-site or third-party sources, supporting connections between industries, like mining, and communities. Reclaimed water can be used for irrigation or industrial processes, including treated municipal wastewater in mining (Decree−Law No. 119/2019, of August 21, 2019). Spain’s Royal Decree, 1620/2007 marked a pivotal step in wastewater reuse, establishing regulations, quality standards, and authorized uses across five sectors: urban, agricultural, industrial, recreational, and environmental. The 2010 Guide for the Application of the Decree provided detailed instructions on maintaining standards and promoting proper reuse. Royal Decree 1085/2024 updated the framework to enhance safety, environmental and health protection, support the circular economy, and include industrial reuse under the broader concept of water recirculation (Real Decree, 1620 / 2007, of December 7, 2007) (Real Decreto, 1085/2024, de 22 de Octubre, 2024). France introduced Decree 2023–835/2023 concerning the uses and conditions for the use of rainwater and treated wastewater. The primary objective of this decree is to define the conditions for the use of rainwater for non-domestic purposes (Décret No 2023–835, 2023) The Decree was further refined by two subsequent orders, published on December 14 and 18, 2023, which established specific conditions for the production and use of treated wastewater in watering green spaces and agricultural crop irrigation. In Italy, the Decree-Law 39/2023 presents urgent provisions to combat water scarcity and to strengthen and adapt water infrastructure (Decreto-Legge39/, 2023, 2023). Art. 5 ensures water availability for irrigation, human consumption, industries and power generation.

Overall, the European regulatory framework demonstrates a clear predominance of agricultural irrigation as the primary and most technically structured application of water reuse. Although industrial reuse is formally acknowledged in policy documents and strategic communications, specific physicochemical and operational parameters tailored to industrial processes are generally absent. The regulatory instruments emphasize environmental protection, water quality preservation, and risk prevention, reflecting a precautionary and ecosystem-oriented approach. Consequently, the framework tends to be preventive in nature, focused on safeguarding receiving bodies and public health, rather than operationally prescriptive for sector-specific reuse applications such as industrial or mining activities. This imbalance suggests that, while reuse is institutionally encouraged, practical implementation in industrial contexts may require supplementary technical criteria beyond those currently defined at the European level.

Since 2010, the Government of Canada has been updating the Canadian Guidelines for Domestic Reclaimed Water for Use in Toilet and Urinal Flushing (Canadian Guidelines for Domestic Reclaimed Water for Use in Toilet & Urinal Flushing, 2010) with the last version published in 2023. Those guidelines explicitly state that “all domestic reclaimed water used for toilet and urinal flushing should be disinfected”, which could occur through chemical, physical or biological processes, in addition to chlorination. This is the only guidance related to water reuse, as there is no federal policy in Canada, which could be applied in mining companies, since its application in toilet and urinal flushing can significantly reduce freshwater consumption in daily industrial routines. In the United States, the Environmental Protection Agency (EPA) launched the Guidelines for Water Reuse in September 2012 (U.S. Environmental Protection Agency, 2012). This document has a structure divided into nine chapters, and it aims to detail technologies aspects, while including the concept of holistic water management by incorporating nonconventional water sources into comprehensive water management planning. Within the urban reuse category, the guideline specifies criteria for applications such as golf course and recreational field irrigation. Despite its significance, the document has not undergone a direct review to date. This is largely due to the legislative framework, which permits each state to develop and implement its own regulations, an approach that also extends to water reuse standards. The Brazilian regulatory scenario contrasts with that of countries where water scarcity has historically driven the consolidation of comprehensive reuse frameworks. Unlike arid and semi-arid nations such as Israel, Jordan, or the United Arab Emirates, where reuse is embedded as a structural pillar of national water security, Brazil possesses extensive surface and groundwater resources, including large river basins and significant aquifer systems. This relative hydrological abundance has historically reduced the urgency of developing detailed and operationally robust reuse regulations at the federal level. The only normative in Brazil about regulation of WR in the national field is Resolution n. 54, November 28, 2005 (RESOLUÇÃO No 54 & DE 28 DE NOVEMBRO DE, 2005, 2005). It establishes modalities, guidelines and general criteria for the practice of direct non-potable water reuse and provides other measures.

In Egypt, effluent reuse for industrial purposes remains limited, as many industrialists are concerned about the potential negative effects of treated wastewater on machinery. However, the enforcement of Environmental Law 4/1994 has encouraged industries to treat and reuse their effluents whenever feasible (The Environmental Law, 1994). Australia is a key reference in water reuse, with the 2006 National Water Quality Management Strategy including guidelines for recycling in industrial uses like cooling, process, and washdown water. It also addresses fire control, dust suppression, toilet flushing, street cleaning, car washing, and irrigation, focusing on health and environmental safety (Australian Guidelines for Water Recycling: Managing Health & Environmental Risks, 2006).

When compared to the broader European Union framework, those national regulations demonstrate greater contextual adaptation and, in some cases, more explicit authorization procedures. However, similar to the EU-level legislation, those countries prioritize irrigation-oriented standards and an environmental protection rationale centered on safeguarding public health and receiving bodies. Industrial reuse, although increasingly acknowledged, often lacks the same degree of parameter specificity and operational guidance. This indicates that, across both supranational and national levels, regulatory evolution has been driven primarily by agricultural and environmental concerns, with industrial applications developing in a more incremental and less technically standardized manner.

Jordan, among the most water-scarce countries, makes water reuse central to its national strategy. Policies like the Water Substitution and Reuse Policy and “Moving from Theory to Action” promote treated wastewater use in irrigation, freeing freshwater for cities and enabling reuse in other economic sectors (Tarawneh et al., 2024). In the same region, Israel stands as a global pioneer in water reuse. The establishment of a comprehensive wastewater treatment and reuse program as a national policy initiative in Israel dates to 1959, through Water Law. This initiative incorporated not only large-scale treatment and reuse measures but also the development of appropriate procedures to ensure adequate risk assessment of associated environmental impacts (Arlosoroff, 2007). As a result, more than 80% of wastewater is reclaimed and reused across multiple sectors, including agriculture, landscaping, and industry (Ministry of Health, 2023). Singapore has implemented the reuse of treated municipal wastewater for non-potable industrial purposes through its advanced treatment scheme known as NEWater (PUB, 2022). This process involves a multi-barrier approach comprising three sequential stages: (i) microfiltration or ultrafiltration, (ii) reverse osmosis, and (iii) ultraviolet (UV) disinfection, which collectively ensure the production of high-quality reclaimed water.

In these cases, severe water scarcity operates as the primary structural driver for regulatory development and policy innovation. Water reuse is not treated as a complementary environmental measure, but rather as a foundational component of national water security strategies. In Jordan and Israel, reuse policies are deeply embedded in long-term resource planning, enabling the strategic reallocation of freshwater to priority sectors while integrating treated wastewater into agricultural, industrial, and urban systems. The institutionalization of reuse through dedicated legislation, centralized oversight, infrastructure investment, and risk management procedures demonstrates a high level of governance maturity aligned with hydrological necessity.

Collectively, these regulatory models reveal a direct correlation between hydrological stress and regulatory comprehensiveness. In water-scarce contexts, reuse becomes a structurally embedded policy instrument, integrated into national security planning and economic development strategies, rather than merely an environmental compliance mechanism.

**Fig. 1:**
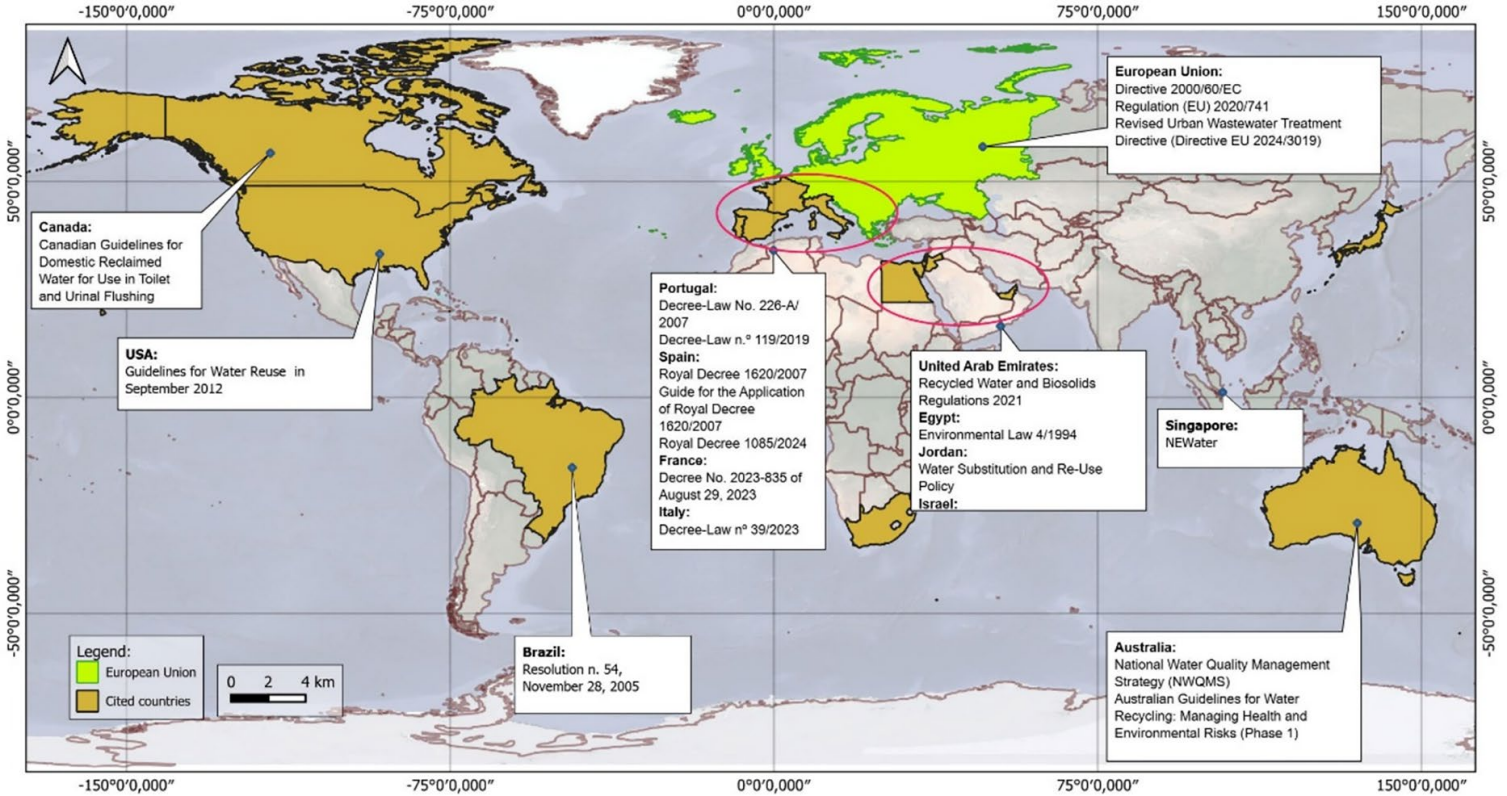
Regulatory frameworks for wastewater reuse worldwide. *Source*: Authors (2026)

Although Ireland, Finland, Lithuania, Latvia, Poland, Austria, the Czech Republic, and Slovakia are developed countries, they do not currently permit the use of reclaimed water for plant irrigation (Directive EU 2024/3019, 2024). This illustrates that, despite technological and regulatory advances in water reuse, some governments remain reluctant to adopt this alternative source. Figure 1 presents the regulatory frameworks for wastewater reuse cited in this study.

The interaction between the water quality characteristics identified in Table 3 and the regulatory landscapes reviewed above creates a direct “regulatory nexo”. For instance, the high metallic concentrations (e.g., Fe up to 2230 mg/L in AMD) trigger more complex environmental licensing and legal liability than the reuse of municipal effluent, which follows established secondary treatment standards. In the proposed framework, this is operationalized through three indicators for Dg:

- Regulatory compatibility (Creg): Evaluates if the intended quality (based on Table 3) is legally permissible for the specific mining end-use. A score of 0.0 acts as a structural veto, indicating that current laws prohibit such reuse regardless of technical feasibility.
- Institutional alignment (Ainst): Measures the maturity of the legal framework (e.g., scoring higher in Spain or Israel than in regions with fragmented or absent reuse policies).
- Licensing complexity (Lcomp): Assesses the administrative time and risk of litigation, which is typically higher for treated AMD reuse than for external municipal supply.

In summary, the regulatory landscape for water reuse reveals both convergence and fragmentation at the international level. A common element across most jurisdictions is the recognition of water reuse as a strategic instrument to enhance water security, reduce pressure on conventional freshwater sources, and advance circular economy principles. Many frameworks establish formal authorization procedures and assign institutional responsibilities, while prioritizing agricultural irrigation as the primary and most regulated application. Another recurring feature is the emphasis on public health protection and environmental safeguards, often through treatment standards, monitoring requirements, and risk management approaches. However, significant differences persist regarding scope, regulatory detail, and sectoral coverage. This regulatory misalignment not only generates uncertainty for operators but also constrains the scalability of reuse initiatives across jurisdictions. Without clearer integration between reuse legislation and industrial process requirements, technically feasible solutions may remain underutilized. A more harmonized and process-sensitive regulatory approach is therefore necessary to facilitate practical implementation.

## Process compatibility and technical feasibility

Water reuse feasibility in mineral processing is fundamentally determined by the degree of compatibility between water quality and process requirements. Different unit operations exhibit distinct sensitivities to physicochemical parameters, and therefore the technical viability of reuse cannot be assessed solely based on regulatory compliance or generic water standards. Instead, feasibility depends on how specific constituents interact with mineral surfaces, reagents, and equipment under operational conditions.

In the proposed framework, the Technical Feasibility (Dt) is operationalized through three core indicators, bridging the gap between raw water characteristics (Sect. “Mining Wastewater Characteristics”) and operational demand:

- Process compatibility (Cproc): Measures the alignment between the chemical profile of the source (Table 3) and the tolerance of the mineral circuit.
- Treatment effort (Teff): Evaluates the complexity of the technology required to bridge the quality gap (e.g., simple filtration vs. advanced membranes).
- Operational reliability (Rops): Assesses the stability of the water supply in terms of quantity and quality over time.

Washing and gravitational concentration processes generally present lower sensitivity to water composition when compared to chemical-intensive methods. In many cases, strict chemical control is not required, and moderate variations in salinity or dissolved species can be tolerated without compromising recovery rates, a logic that aligns with the criteria-structuring approach for mining-related decision-making which differentiates technical requirements based on specific operational goals (Amaro et al., 2021, 2022). This operational flexibility increases the feasibility of internal recycling and even the use of externally treated effluent. However, when turbidity or specific chemical indicators directly influence product specifications, more rigorous quality control becomes necessary. Even in relatively tolerant systems, dissolved ions may interact with mineral surfaces in suspension, altering physicochemical behavior and potentially compromising product quality.

In contrast, flotation circuits are significantly more sensitive to water chemistry. Parameters such as salinity, electrical conductivity, pH, dissolved oxygen, calcium and magnesium concentrations, and trace metals directly influence reagent adsorption, froth stability, mineral surface charge, and the formation of insoluble complexes. High ionic strength may promote precipitation reactions with fatty acid collectors, while pH variations interfere with froth formation and mineral selectivity (CETEM, 2010). Dissolved oxygen affects the process because it controls the mineral surface composition (Liu et al., 2013), and variations in pH can interfere with flotation by affecting froth formation,since pH-dependent dissolution and oxidation reactions can generate ionic species (e.g., thiosalts and metal ions) that modify froth stability and gas dispersion characteristics. Elevated temperatures can increase the flotation rate by shortening residence time and improving sulfide grade; conversely, they can also increase reagent solubility and mineral dispersion. Experimental results show that increasing temperature from approximately 20–60 °C can enhance flotation rates, reducing the required residence time by up to 30–40% while improving sulfide mineral grades due to faster collector adsorption and bubble–particle attachment (Bicak et al., 2023). These interactions demonstrate that water reuse in flotation is conditionally feasible and requires prior physicochemical validation.

For the indicator Cproc, the framework applies a threshold logic:

- Score 1.0 (High): When water quality (e.g., treated municipal effluent) requires only minor adjustments (pH/solids) to meet flotation standards.
- Score 0.5 (Conditional): When high ionic loads or specific ions (e.g., sulfates in AMD) require advanced treatment to prevent metallurgical loss.
- Score 0.0 (Veto): When a ‘Technical Veto’ is triggered. For instance, if residual reagents or extreme salinity would irreversibly impair mineral recovery, regardless of treatment cost.

Quality must therefore be defined according to the operational tolerance of each unit process and reagent system. Ideally, these parameters should be established through laboratory and pilot-scale testing, allowing adjustment of water conditions to simulate full-scale operation (IBRAM & ANA, 2006). Without this compatibility assessment, the integration of reused water may compromise equipment integrity, reduce mining recovery, and increase overall costs, potentially offsetting the environmental and economic benefits of reuse.

One of the major operational challenges in mining is the increasing reliance on impure primary water sources and the progressive accumulation of dissolved species in recirculated water from tailings ponds, thickener overflows, and filtration systems. These streams may contain elevated salinity, calcium, magnesium, iron, and residual organic reagents such as depressants, collectors, dispersants, and flocculants. The buildup of these constituents can negatively affect flotation efficiency, increase reagent consumption, promote scaling and corrosion, and elevate operational costs (CETEM, 2001).

In copper mining, water recirculation without correct treatment led to significant copper loss to the pre-flotation concentrate, indicating that untreated effluent negatively impacts flotation efficiency (Ozturk et al., 2022). Treating the recirculated water with activated carbon restored flotation efficiency by preventing the loss of copper and zinc to the pre-float section (Ozturk et al., 2022). The presence of natural organic matter in reclaimed water can alter the behavior of dissolved organic carbon, impacting reagent interactions (Drewes & Fox, 2000). These trends indicate that reuse feasibility is strongly site- and ore-dependent.

Overall, the data indicate that reuse feasibility can be hierarchically classified as exemplified below:

- Higher feasibility: coal mining waters and low-sensitivity process streams after basic treatment.
- Conditional feasibility: selected gold mining effluents requiring targeted physicochemical correction.
- Low feasibility without advanced treatment: copper and pyrite acid drainage streams with high acidity, salinity, and metal loads.

In this hierarchy, treated municipal wastewater consistently scores higher in Dt (typically 1.0 or 0.5) than internal AMD streams (typically 0.0 or 0.5) due to its lower mineralization and more manageable chemical profile, as contrasted in the comparative analysis below. Importantly, regulatory compliance alone is insufficient to determine suitability for reuse in mineral processing. Operational compatibility, particularly with flotation chemistry, must be verified through pilot-scale testing, as ionic strength, calcium concentration, sulfate levels, and residual metals directly influence reagent performance and metallurgical recovery. Based on that, it is then possible to identify and recommend specific operations that could prioritize the use of internally or externally reused water. For example, processes that require water with low salinity could benefit from the use of domestic treated wastewater, which generally exhibits low salinity levels. This approach not only facilitates the treatment process but also promotes more efficient and sustainable water management practices within the mining sector.

Industrial case studies reinforce this conclusion. The use of neutralized AMD as a replacement for potable water as make-up water during the flotation of a platinum-group-minerals (PGM)-bearing Merensky ore was the target of research (Taguta et al., 2022). The results demonstrated that water quality strongly affects flotation performance. Under standard depressant dosages (50 g/t), AMD neutralized with Ca(OH)₂ yielded significantly lower PGM recoveries compared to potable water, with rougher recovery decreasing from 88.4 (potable water) to 67.8%, and cleaner recovery dropping from 84.9 to 58.6%. Treatment using the Veolia process reduced sulfate concentrations by approximately 90%, from 2730 to 109 ppm; however, this was accompanied by a substantial increase in calcium (from 667.5 to 1202 ppm), chloride (from 66.1 to 481.6 ppm), and ionic strength, as indicated by the rise in conductivity from 3600 to 9350 µS/cm. Although this treatment improved flotation performance relative to untreated AMD, the PGM rougher recovery (70.8%) remained below that achieved with potable water. These results indicate that, despite effective sulfate removal, the increased ionic strength and altered ionic composition constrained recovery improvements. This case highlights the potential of AMD reuse as a strategy to reduce freshwater consumption while simultaneously mitigating environmental liabilities associated with mine drainage, reinforcing the role of water recycling and reuse as key components of sustainable mining operations.

Another case comes from Rosh Pinah mine in Namibia, where water is recovered from the lead rougher tailings thickener and the lead concentrate thickener, and subsequently reused in the milling and lead flotation processes (Seke & Pistorius, 2006). The chemical composition of this recycled water includes significant concentrations of dissolved species, such as Cu (17.2–30 mg/L), Zn (0.34–10 mg/L), Pb (0.33–0.5 mg/L), and free cyanide (53–60 mg/L), with total dissolved solids reaching up to 949 mg/L. These species were shown to directly affect flotation selectivity through electrochemical mechanisms. In particular, variations in pulp electrochemical potential (Eh) of approximately 260 mV were observed between wet and dry milling conditions, significantly influencing mineral surface reactions. These findings demonstrate that flotation selectivity in complex sulfide ores is strongly dependent on the electrochemical potential of the pulp, which is, in turn, controlled by the composition of recycled process water and operational conditions (Seke & Pistorius, 2006).

The application of ion-exchange resins for process water treatment in an Iberian Cu–Zn ore plant highlights its role as a strategy for water reuse in mining operations. Laboratory tests and plant validation showed that strong base-type resins, converted from Cl⁻ to OH⁻ form were able to reduce sulfate concentrations in flotation water by approximately 75–85%, depending on operating conditions, with residual sulfate levels below 500 mg/L under optimized scenarios (Can et al., 2020). Importantly, the ion-exchange resin could be regenerated with NaOH solution and reused multiple times, reinforcing its feasibility as a sustainable water treatment method. The findings underline that process water chemistry is site-specific, requiring optimization of flow rates and contact times for effective treatment. As such, this case illustrates a concrete example of water reuse in mining, showing how partial cleaning of circuit water can reduce freshwater demand, mitigate the negative impacts of recirculated ions on flotation, and promote more sustainable water management practices in mineral processing.

To ensure safety and operational reliability, NEWater is subject to stringent annual audits conducted by national authorities and independent international experts, consolidating its position as a global reference in advanced wastewater reclamation. Most NEWater is supplied to industrial users through a dedicated distribution system for non-potable purposes, while a smaller fraction is blended with raw water in reservoirs. The proportion directed to reservoirs is flexible and strategically increased during dry periods to enhance water security. Unlike regulatory frameworks in some countries that classify reclaimed water according to its intended use, Singapore does not assign specific categories to treated municipal wastewater used in industrial applications. Instead, NEWater is regarded as high-grade reclaimed water of sufficient quality to meet potable standards (EPA, 2023).

When compared to AMD and wastewater from mining operations, raw domestic sewage presents a substantially different and more manageable profile in terms of water quality and treatment requirements. According to von Sperling (2011) typical values for raw domestic sewage include total solids (TS) ranging from 700 to 1300 mg/L, alkalinity between 100 and 250 mg CaCO₃/L, ammoniacal nitrogen (N–NH_4_⁺) from 20 to 35 mg/L, total Kjeldahl nitrogen (TKN) between 35 and 60 mg/L, orthophosphate (P–PO_4_^3^⁻) from 3 to 9 mg/L, and pH values typically in the 6.7–8.0 range.

This composition is considerably more favorable for treatment than mining effluents, which often display extreme pH (as low as 1.3), high electrical conductivity (often > 10,000 µS/cm), heavy metal concentrations exceeding hundreds or thousands of mg·L-1, and very high levels of sulfates and total dissolved solids (TDS). Moreover, while domestic sewage is organic and biodegradable, requiring microorganism removal, mining effluents are characterized by inorganic pollutants, particularly metals and acid-generating compounds, which are non-biodegradable and more recalcitrant in treatment systems.

Domestic wastewater may typically be treated through conventional biological processes (e.g., activated sludge, stabilization ponds, anaerobic reactors), which are both cost-effective and robust, especially in developing regions. When low-salinity water is needed, even sewage needs to be treated by advanced processes such as Reverse Osmosis. However, given the lower salinity, the process will be less energy-intensive compared to treating mining effluent. In contrast, mining waters often require advanced physicochemical treatments, including lime neutralization, chemical precipitation, oxidation, ion exchange, and membrane filtration, which are costly, energy-intensive, and operationally demanding.

The buffering capacity of domestic sewage is higher due to its moderate alkalinity, which facilitates pH control in biological reactors. Mining effluents, especially those from pyrite and gold mining, may contain sulfuric acid and extremely low alkalinity, leading to acidic shocks and requiring external alkaline addition for any biological or chemical treatment to proceed.

## Economic feasibility

The economic feasibility of wastewater reuse in mining is highly context-dependent and cannot be generalized across technological configurations. Comparative assessments of centralized and decentralized systems show inconsistent results: while some studies report lower material, energy, and greenhouse gas (GHG) impacts for centralized systems, others indicate reduced energy use and emissions in decentralized arrangements. This variability highlights the influence of technological design, political and social settings, and topographical conditions on system performance (Sgroi et al., 2018).

To transform this multidimensional variability into a reproducible decision-support tool, the De dimension is operationalized through three normalized indicators:

- Unit cost ratio (Cunit): Compares the levelized cost of reclaimed water (including treatment and conveyance) against the cost of the current primary water source (freshwater or desalination).
- Infrastructure intensity (Iinf): Evaluates the CAPEX burden required for dedicated conveyance (pipelines and pumping stations), considering the spatial mismatch between sources.
- Economic resilience (Recon): Assesses the long-term protection against water-related financial risks, such as scarcity-induced production halts or rising abstraction fees.

Despite technological maturity, reuse remains limited due to institutional, social, and economic barriers (Sgroi et al., 2018). In mining regions, these constraints are compounded by high volumetric demand, spatial separation between wastewater treatment plants and mining facilities, and the need for dedicated conveyance infrastructure.

Economic assessment must therefore adopt a systemic and lifecycle perspective. Reuse projects should be evaluated through whole-life benefit–cost analysis, incorporating planning, design, construction, operation, maintenance, and decommissioning phases (Wilcox et al., 2016). Short-term cost comparisons are insufficient to capture infrastructure depreciation, long-term liabilities, and operational risk.

Market conditions further complicate economic comparison. Variability in local construction costs, regulatory requirements, and treatment technologies limits cross-regional benchmarking. However, unit cost metrics enable comparison with alternative water supply options such as desalination, indirect or direct potable reuse, and non-potable reuse (Nasiri et al., 2013). In water-scarce mining regions, increasing freshwater abstraction costs may shift the relative competitiveness of reclaimed water.

System configuration and scale are critical determinants of cost structure. Capital expenditures (CAPEX) typically include pipeline infrastructure, pumping stations, equalization reservoirs, polishing systems (where required), and modifications to industrial processing circuits. Operational expenditures (OPEX) are largely driven by pumping energy (which is strongly dependent on distance and elevation gradients), chemical inputs, and continuous quality monitoring. Furthermore, OPEX includes pipeline maintenance (e.g., corrosion and scaling control) and risk management measures, such as cross-connection and backflow prevention. These factors influence centralized and decentralized systems to a greater or lesser degree. Centralized systems may benefit from economies of scale in treatment, yet often require substantial capital and energy investment in long-distance conveyance. Conversely, decentralized systems can reduce distribution requirements but may increase per-unit treatment costs. The optimal configuration is therefore site-specific and conditioned by hydraulic constraints, infrastructure compatibility, and demand concentration.

The framework applies a scoring threshold to determine financial viability:

- Score 1.0 (High): Reclaimed water cost is lower than or equal to current freshwater costs, or the distance to the source is minimal (< 10–20 km).
- Score 0.5 (Conditional): Costs are higher than conventional sources, but justified by long-term water security or ‘Environmental, Social, and Governance’ (ESG) gains.
- Score 0.0 (Veto): Triggered when the ‘Economic Veto’ is met. When CAPEX for conveyance or OPEX for advanced treatment (e.g., for extreme AMD) makes the project financially ruinous, exceeding the mine’s operational margin.

From a treatment standpoint, municipal wastewater is generally less complex and less costly to treat than AMD or metal-laden mining effluents. Domestic sewage is predominantly organic and biodegradable, allowing the application of conventional biological processes with comparatively moderate energy and chemical requirements (von Sperling, 2011). In contrast, AMD typically demands continuous neutralization, chemical precipitation, sludge handling, and, in some cases, advanced polishing processes, resulting in higher reagent consumption, energy demand, and operational complexity (Masindi et al., 2022). Under certain conditions it may be economically rational to utilize treated municipal effluent as an alternative supply rather than expanding internal AMD treatment capacity.

However, domestic wastewater is commonly produced in centralized municipal wastewater treatment plants located near urban centers rather than in proximity to mining operations. This spatial mismatch may impose constraints related to conveyance infrastructure, pumping energy, elevation differences, and associated capital and operational costs. For large-scale mining projects situated in remote areas, transporting reclaimed water over long distances can significantly influence overall economic and environmental feasibility. Therefore, beyond compliance with water quality standards and volumetric adequacy, spatial configuration and infrastructure requirements become critical determinants of practical implementation.

Consequently, the relative economic advantage of domestic municipal wastewater versus expanded on-site process effluent treatment is highly site-specific. Where mines are located within reasonable distance of urban treatment plants and topographic conditions are favorable, reuse from domestic municipal wastewater may present a competitive and strategically resilient supply option. Conversely, in remote settings with significant elevation gradients or long-distance transport, the cost of conveyance may offset treatment savings. Economic feasibility thus depends on a balanced assessment of treatment intensity, infrastructure requirements, hydraulic constraints, and long-term water security considerations. Given these interdependencies, no single technological or financial arrangement can be universally prescribed (Wilcox et al., 2016), reinforcing the need for an integrated decision-support framework capable of aligning economic, technical, and governance dimensions.

## Integrated framework for water reuse feasibility in mining

**Fig. 2:**
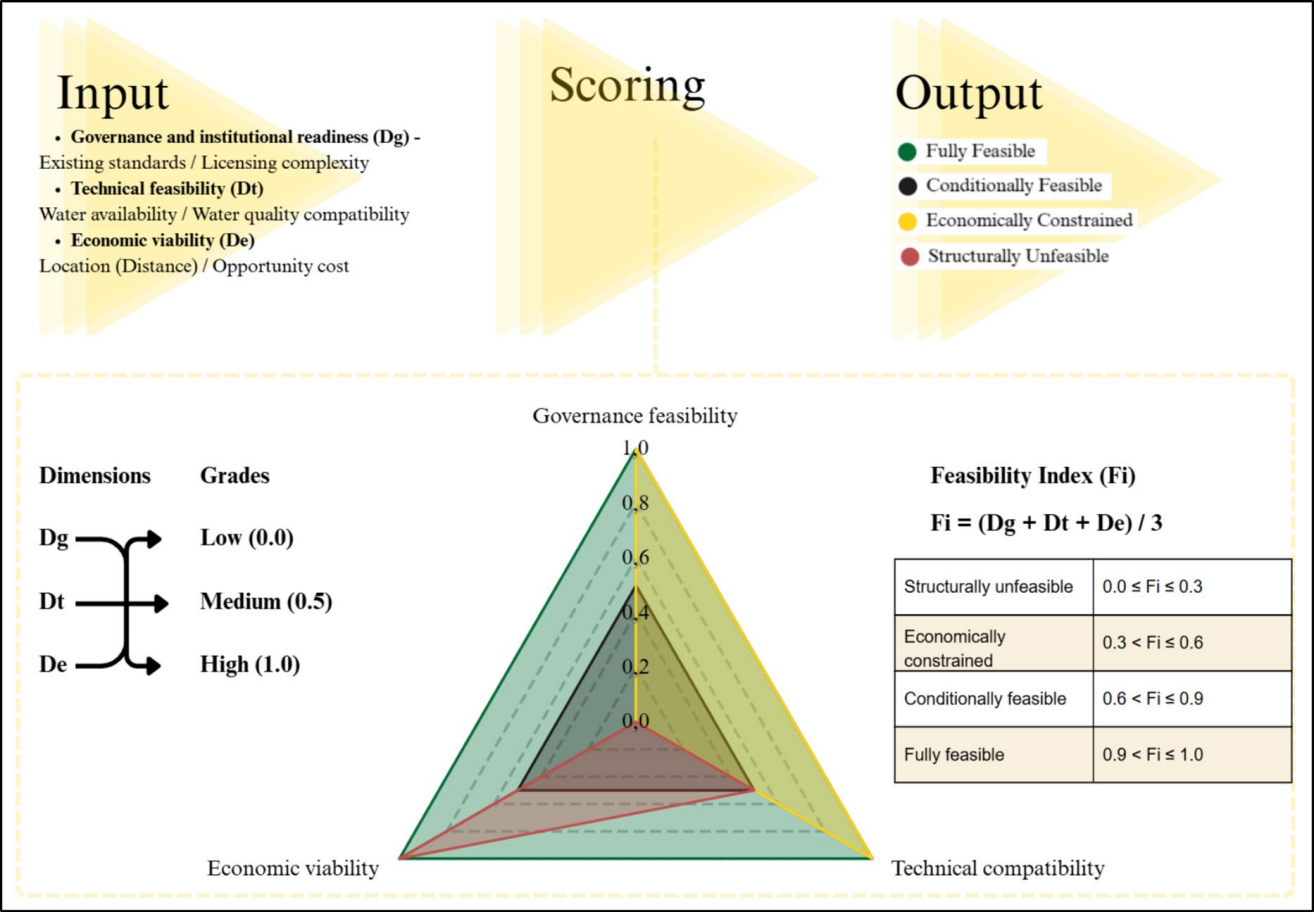
Operational MCDM framework for feasibility classification of wastewater reuse in mining. *Source*: Authors (2026)

This study proposes an integrated decision-support framework to evaluate the feasibility of reclaimed water use in mineral processing (Fig. 2). The framework consolidates three interdependent dimensions: (i) governance and regulatory alignment, (ii) process compatibility and technical feasibility, and (iii) economic viability. Rather than treating these dimensions independently, the framework conceptualizes feasibility as a systemic condition emerging from their interaction. Water reuse in mining is therefore defined not merely as regulatory compliance or cost competitiveness, but as the convergence of institutional, technical, and economic viability within a specific spatial and operational context.

The four outcome categories derived from the proposed framework can be illustrated through representative hypothetical scenarios. A fully feasible case would involve a mining operation located within moderate proximity to a municipal wastewater treatment plant, where reclaimed water meets regulatory standards and exhibits physicochemical characteristics compatible with the mineral processing circuit, particularly low sulfate concentration, moderate hardness, and stable ionic composition. In such a scenario, governance structures are clearly defined, industrial reuse is legally authorized, monitoring protocols are established, and the required infrastructure (pipeline and pumping system) results in lifecycle costs competitive with alternative water supply options. Under these conditions, institutional alignment, operational stability, and economic competitiveness converge, enabling immediate implementation with minimal system adaptation.

A conditionally feasible scenario would occur when regulatory authorization is present and infrastructure costs are manageable, but technical compatibility requires targeted adjustments. For example, reclaimed municipal effluent may comply with microbiological and conventional parameters yet present elevated sulfate or hardness levels that interfere with flotation selectivity. In this case, feasibility would depend on additional polishing treatment, blending strategies, or circuit modifications to prevent recovery losses or reagent overconsumption. Implementation remains viable, but a pilot-scale validation and operational optimization to mitigate identified technical risks would improve realistic results.

An economically constrained case would emerge when governance and technical compatibility are satisfactory, yet spatial configuration renders the project financially unattractive. This situation may arise when a large-scale mining operation is located far from centralized wastewater infrastructure, requiring long-distance conveyance and high-energy pumping due to elevation differences. Even if reclaimed water meets process requirements and regulatory conditions are favorable, excessive capital expenditures for pipeline construction and sustained operational energy costs may exceed the economic threshold relative to alternative water sourcing or internal treatment expansion. Here, feasibility is technically sound but financially unsustainable under prevailing cost structures.

Finally, a structurally unfeasible scenario would be characterized by fundamental governance or technical barriers that prevent implementation irrespective of economic considerations. This could include jurisdictions where industrial reuse is not legally authorized or where regulatory frameworks lack clarity regarding liability and compliance responsibilities. One example is a context in which treated municipal wastewater plays a critical role in maintaining regional water security, being the effluent currently used for groundwater recharge and supports agricultural activities that are highly dependent on water reuse, particularly in regions affected by chronic water scarcity. This type of allocation conflict is commonly observed in water-scarce regions, where reclaimed water is strategically reserved for agriculture and aquifer recharge. In such cases, one or more critical dimensions of the framework approach a structural constraint threshold, rendering the reuse strategy impracticable under current institutional and operational conditions.

To operationalize the proposed framework and demonstrate its practical utility as a decision-support tool, a pseudo-application was conducted using three contrasting hypothetical scenarios. These scenarios utilize the real water quality baseline data previously presented in Table 3 and simulate typical implementation challenges in the mining sector. The scenarios are defined as follows in Table 4.

**Table 4:** Worked example: operationalizing the framework across three contrasting scenarios

	Scenario A: municipal supply (Nearby)	Scenario B: municipal supply (Remote)	Scenario C: internal AMD reuse
Dimension / Sub-criteria	A gold mining operation located 8 km from an urban WWTP. The jurisdiction has specific industrial reuse regulations (e.g., Spain or Israel). The reclaimed water requires only minor polishing to match flotation requirements	A copper mining operation located 60 km from the nearest urban WWTP, with significant elevation gradients. While the governance and technical compatibility are favorable, the spatial mismatch is severe	A remote copper mine attempting to reuse its own AMD in the flotation circuit (pH 2.98, Cu 398.2 mg/L, Fe 2230.6 mg/L, from Table 3)
Governance (Dg)			
Existing standards	1.0 (Specific legislation exists)	1.0 (Specific legislation exists)	0.5 (General norms/Complex discharge)
Licensing complexity	1.0 (Simplified process)	0.5 (Complex due to 60 km pipeline)	0.0 (High risk/Community rejection)
**Dg average score**	**1.00**	**0.75**	**0.25**
Technical (Dt)			
Water availability	1.0 (Continuous supply > 70%)	1.0 (Continuous supply > 70%)	1.0 (Continuous internal supply)
Water quality compatibility	0.5 (Requires minor polishing)	0.5 (Requires minor polishing)	0.0 (Contaminants degrade process)*
**Dt average score**	**0.75**	**0.75**	**0.50 (Veto triggered)**
Economic (De)			
Location (Distance)	1.0 (< 10 km)	0.0 (> 50 km, prohibitive CAPEX)	1.0 (Internal, 0 km)
Opportunity cost	1.0 (Cheaper than freshwater)	0.0 (Pumping OPEX exceeds freshwater)	0.0 (Treatment OPEX is prohibitive)
**De average score**	**1.00**	**0.00**	**0.50**
**Feasibility Index (Fi)**	**0.91**	**0.50**	**0.41 (Downgraded)**
**Final Classification**	**Fully Feasible**	**Economically Constrained**	**Structurally Unfeasible**

In Scenario C, the extreme metallic load of the AMD directly impairs flotation, yielding a 0.0 in quality. Furthermore, an economic score of 0.0 in Scenario B and licensing/quality scores of 0.0 in Scenario C activate the framework’s veto logic. The categories grades (average score) and final classification are in bold

As demonstrated in Table 4, the framework successfully differentiates the operational realities of each project. Scenario A achieves a Fi of 0.91, indicating that treated municipal wastewater is an excellent strategic choice when spatial and regulatory alignments are favorable. Conversely, Scenario B demonstrates how a project with perfect technical compatibility (Dt = 0.75) can be rendered *Economically Constrained* (Fi = 0.50) due exclusively to the geographical distance (> 50 km) and the associated infrastructure CAPEX, validating the framework’s multidimensional sensitivity. Finally, Scenario C highlights the critical importance of the ‘veto logic’. Despite the zero distance (Economic sub-score 1.0), the extreme chemical complexity of the copper AMD (Technical sub-score 0.0) and the high licensing risk (Governance sub-score 0.0) trigger structural vetoes. Even with a mathematical average of 0.41, the project is correctly downgraded to *Structurally Unfeasible*, proving that internal recycling of highly degraded mine-affected waters is often inferior to sourcing external municipal effluents. Conversely, when the WWTP is located at a greater distance from the mine, the feasibility of water reuse becomes constrained by increased conveyance costs and infrastructure requirements. In such cases, even if the treated wastewater meets quality standards, the economic advantage may be offset by transport limitations, potentially leading to supply shortages. As a result, locally available AMD (although characterized by higher salinity) may become a more cost-effective option due to reduced logistical constraints.

While the proposed integrated framework provides a novel and structured approach to assessing water reuse feasibility in mining, it is important to acknowledge its current limitations, which serve as a roadmap for future research. First, the present model utilizes an unweighted Multi-Criteria Decision-Making approach, assigning equal importance to the Technical, Regulatory, and Economic dimensions. While this ensures a balanced baseline assessment, in specific regional contexts, one dimension may exert a disproportionate influence on the final decision (e.g., in regions with extreme scarcity, the economic dimension may be less restrictive than the technical one). Future refinements may incorporate techniques to elicit stakeholder preferences and assign dynamic weights that reflect local priorities and expert elicitation. Second, the scoring matrix currently employs a three-point ordinal scale (0, 0.5, 1.0) to maintain simplicity and ease of application during early-stage screening. However, certain technical parameters, such as the specific hydrochemical compatibility for flotation, may require greater range. Future iterations of the tool could benefit from a five-point or continuous scale, potentially linked to specific unit treatment costs or mineral recovery thresholds, providing more precise sensitivity analysis. Lastly, although the framework incorporates licensing and governance, the current MCDM structure lacks explicit multi-stakeholder preference elicitation for criteria weighting. Future iterations should incorporate participatory weighting techniques, such as the Analytic Hierarchy Process (AHP) or the Simple Multi-Attribute Rating Technique Extended to Ranking (SMARTER) (Amaro et al., 2022), to directly embed community and expert values into the decision matrix. Additionally, integrating AI-driven narrative analysis could provide cross-jurisdictional insights into how SLO and ESG risks evolve, allowing the framework to capture shifting social perceptions, Indigenous rights, and distributional fairness in real-time (Rodolaki et al., 2025). Addressing these uncertainties and embedding social risk metrics will be essential to evolve this conceptual model into a high-precision, life-cycle decision-support sys.

## Conclusion

This study developed and operationalized an integrated analytical framework to assess the feasibility of treated municipal wastewater reuse in mining operations. By structuring regulatory compliance, technical compatibility, and economic viability within a purpose-built Multi-Criteria Decision-Making approach, the proposed model moves beyond fragmented assessments that traditionally evaluate these dimensions in isolation. A key distinction of this research is the introduction of a ‘veto logic’ within a tridimensional feasibility space, allowing for the systematic identification of critical barriers that mathematical averages often mask.

Through the proof-of-concept application, the framework proved capable of classifying reuse scenarios into fully feasible, conditionally feasible, economically constrained, or structurally unfeasible outcomes. In doing so, the study bridges the gap between water governance, mineral processing sensitivity, and infrastructure planning, offering a reproducible tool capable of supporting strategic resource allocation in water-intensive mining contexts.

Beyond its methodological contribution, the framework provides practical implications for policymakers, regulators, and industry stakeholders seeking to expand circular water strategies under increasing hydrological stress. By explicitly integrating spatial configuration, process-level water quality requirements, and cost determinants, the approach facilitates more realistic assessments of reuse potential, reducing uncertainty in long-term planning. The comparative analysis specifically highlights that municipal effluent often presents superior technical and regulatory viability compared to internal AMD recycling, provided that spatial constraints are managed.

Despite the proposed framework’s robustness as a screening tool, it has limitations that define a clear path for future research. Subsequent studies should explore dynamic weighting methods to address regional priorities and transition toward higher-granularity scales for technical parameters. Furthermore, integrating the Social License to Operate and seasonal climate risks will be essential to evolve this conceptual model into a high-precision decision-support system, capable of addressing the full complexity of water reuse in the global mining industry.

## Competing Interests

The authors declare no competing interests.

## Data Availability

Data will be made available on request.
